# The Preparation and Performance Study of Polyamide Film Based on PDA@MWCNTs/PVDF Porous Support Layer

**DOI:** 10.3390/molecules29071460

**Published:** 2024-03-25

**Authors:** Zhenzhen Xu, Quanjun Li, Xuzhi Sun, Jian Xing, Xinghua Hong, Feng Liu

**Affiliations:** 1 School of Textile and Garment, Anhui Polytechnic University, Wuhu 241000, China; xuzhenzhen@ahpu.edu.cn (Z.X.); 18256794643@163.com (Q.L.); issxz657174979@gmail.com (X.S.); xingjian@ahpu.edu.cn (J.X.); 2 Key Laboratory of Intelligent Textile and Flexible Interconnection of Zhejiang Province, Hangzhou 310018, China; 3 Advanced Fiber Materials Engineering Research Center of Anhui Province, Anhui Polytechnic University, Wuhu 241000, China

**Keywords:** PDA@MWCNTs/PVDF support, PA film, permselectivity, stability

## Abstract

It is urgent to develop a polyamide (PA) thin-film composite (TFC) membrane with a new method in this study by designing and constructing a new nanomaterial support layer instead of the conventional support layer. Polydopamine-wrapped single-walled carbon nanotubes (PDA@MWCNTs) as the place of the polymerization reaction can optimize the PA film structure and performance. The resulting composite membrane presents a higher water flux of 15.8 L·m^−2^·h^−1^·bar^−1^ and a rejection rate of 97% to Na_2_SO_4_, simultaneously maintaining this high separation performance in 300 min. It is a new ideal to construct novel support layer by using inorganic nanoparticles and organic polymer nanofiber membranes.

## 1. Introduction

With the development of scientific technology and industrialization, more environmental problems are continuously manifesting [1,2]. For example, the water pollution phenomenon is becoming more and more severe [3]. How to obtain a clean water source is an urgent problem that we face. At present, seawater desalination and wastewater treatment are effective methods to alleviate water scarcity [4]. In all processing methods, membrane separation technology has attracted widespread attention from researchers because of its unique properties [5]. Nanofiltration (NF) and reverse osmosis (RO) membranes are the core aspects of the membrane separation process [6]. The polyamide (PA) thin-film composite (TFC) membrane is the main structural type of NF and RO membranes. A typical PA TFC membrane is fabricated by the interfacial polymerization (IP) of diamine and acyl chloride to form a PA thin film on a porous support layer. With decades of development, PA TFC membranes have been widely applied commercially [7,8]. However, there are still some defects that restrict its further development, such as the trade-off between the rejection and water flux of PA films [4].

A PA TFC membrane with superior water permeate flux while maintaining high salt ion rejection is still the goal pursued by researchers [9,10,11]. Generally, the PA film is mainly responsible for the rejection and water flux of the TFC membrane. Therefore, most researchers have focused on improving the physicochemical properties of the PA active layer through changing reacted monomer types, the interfacial polymerization method, and surface modification [12,13,14]. At present, there are effective methods to improve the water flux of PA films, such as decreasing the thickness of PA films, shortening the distance of the internal water channel, decreasing the transmission resistance, and increasing the new water channel. To sum up, exploring a high degree of crosslinking and ultrathin PA films is the main research direction of researchers [15,16]. Although the active layer determines the permselectivity of the PA TFC membrane, the structure and properties of the porous support layer still have a non-negligible impact on the IP process. The porous support layer is the reaction site of IP, which influences the IP process and further influences the resulting PA film formation and properties [17,18]. In theory, an ideal support layer should have low flexibility, high surface porosity, and an appropriate pore size. The porous ultrafiltration polymeric membrane is the typical support layer, which is fabricated via the phase inversion technique. Recently, some studies revealed that improving the performance of the TFC membrane by embedding nanomaterials into support layer is an effective method. Mohammed et al. [19] used a nanozeolites-modified support layer and investigated the effect of various concentrations on the overall performance of the TFC membrane. Polisetti et al. [20] utilized SiO_2_/TiO_2_ to modify a PAN/PVDF-blended support layer and fabricated a nanofiltration membrane. The TFC membrane with modified support exhibited higher water flux than the control sample. Xia’s group fabricated nanofiltration membranes (NFMs) via interfacial polymerization on polyvinylidene fluoride (PVDF) substrates modified with hierarchical flower-like molybdenum disulfide (HF–MoS_2_). The TFC membrane exhibited higher selective and permeating performance for Na_2_SO_4_ [21]. Moreover, the functional CNTs also is an ideal nanomaterial to modify the support layer.

In spite of the structure exhibiting excellent stability and separation performance, the water permeation flux is low. In the current decade, exploring and designing a new type of porous support layer has become the subject of remarkable research projects in the field of PA TFC membranes [22,23].

In 2015, Livingston’s [24] group constructed a unique support layer and, by controlling the IP reaction condition, fabricated a PA TFC membrane with unprecedented solvent permeance and high rejection. The support layer was composed of cadmium hydroxide (Cd(OH)_2_) nanowires and a polymer ultrafiltration membrane. The ultrathin PA film was formed on the nanowires, and then the nanowires were removed via dissolving. This report opens up a new research direction for the study of support layers. Wang et al. [25] used cellulose nanocrystals to replace Cd(OH)_2_ nanowires as an interlayer to fabricated a PA TFC nanofiltration membrane, which exhibited an ultrahigh water flux at low operating pressure. Meanwhile, it also exhibited an excellent separation ratio for monovalent/divalent ions. Inspired by this new ideal, some inorganic nanomaterials were used as interlayers such as graphene oxide (GO), metal–organic frameworks (MOFs), and covalent organic frameworks (COFs). To sum up, these materials as interlayers can effectively decrease the thickness of PA films and increase water flux.

Inspired by the above, we used a tailor-made nanofiber membrane to replace the conventional support layer. Based on our previous research, we found that a tree-like structure not only can improve the pore structure of the nanofiber membrane but effectively increase the water flux of the PA TFC membrane. The microfiber of tree-like nanofiber membranes combined with PA films forming new water channels is the main reason for the increased water flux of TFC membranes [26]. However, the construction of a tree-like structure has a certain degree of randomness, as well as being limited by harsh experimental conditions. In order to obtain the support layer with a uniform surface pore structure and stable performance. We constructed a double-layer support layer by depositing inorganic nanoparticles on the nanofiber membrane surface, which to provide new ideal designs of PA TFC membranes. Up to now, there are few studies in the literature on carbon nanotubes (CNTs) interlayers. CNTs exhibit a uniform nanoscale size and excellent chemical stability. Therefore, a CNTs interlayer possesses a uniform and small pore size, high porosity, and smooth surface roughness. However, the agglomeration phenomenon is easy to occur on virgin CNTs. It is essential to modify CNTs’ surface, improving the dispersity of CNTs.

In this research, we fabricated polydopamine-wrapped multi-walled carbon nanotubes (PDA@MWCNTs)/polyvinylidene fluoride (PVDF) nanofiber double-layer support layers. PDA@MWCNTs deposited on the PVDF nanofiber membrane surface via the vacuum filtration method. PDA@MWCNTs as a thin interlayer which has a uniform pore size, high surface porosity, optimal surface hydrophilic ability, and a high ratio of interconnected pores. These advantages facilitate the formation of defect-free and ultrathin PA films by adjusting the IP conditions. The resulting PA TFC membrane exhibited an excellent separation performance with a water flux of 15.8 L/(m^2^·h·bar) and a 97% rejection ratio to sodium sulfate (Na_2_SO_4_).

## 2. Result and Discussion

### 2.1. Characteristic of the Structure and Properties of PDA@MWCNTs

**Figure 1:**
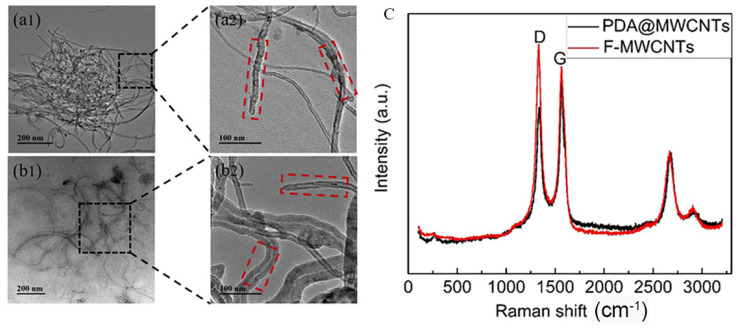
TEM images: (**a1**,**a2**) F-MWCNTs (the black squares for local amplification), (**b1**,**b2**) PDA@MWCNTs (the red squares is the surface morphology of MWCNTs ); (**c**) Raman spectrum of F-MWCNTs and PDA@MWCNTs.

MWCNTs with nanoscale size experience an easy-to-occur agglutination phenomenon in solution. In order to improve the dispersity of MWCNTs, we utilized strong hydrophilic PDA coated on the surface of MWCNTs. The charge effect between the solution and PAD@MWCNTs is conducive to PAD@MWCNTs being uniformly dispersed in water solutions. The microstructure and morphology of modified and unmodified MWCNTs are exhibited in Figure 1a,b. TEM images present unmodified MWCNTs stacked together as a small ball. The PDA@MWCNTs did not appear to experience obvious agglutination and uniformly dispersed on the Cu net. Furthermore, we can observe the obvious structure change between them: the PDA@MWCNTs surface has a significant PDA cladding layer with a smoother surface.

To further prove the structure change, the MWCNTs and PDA@MWCNTs were analyzed via Raman spectroscopy, and the results are shown in Figure 1c. They possess two obvious peaks in the image: the D peak at 1352 cm^−1^ and the G peak at 1750 cm^−1^ [27,28]. Compared the two peak values, we can find that the D peak value of PDA@MWCNTs is smaller, and the G peak value is larger. It indicates that PDA optimized the defect of acid-treated MWCNTs surface.

### 2.2. The Morphology and Properties of PDA@MWCNTs/PVDF Support Membrane

**Figure 2:**
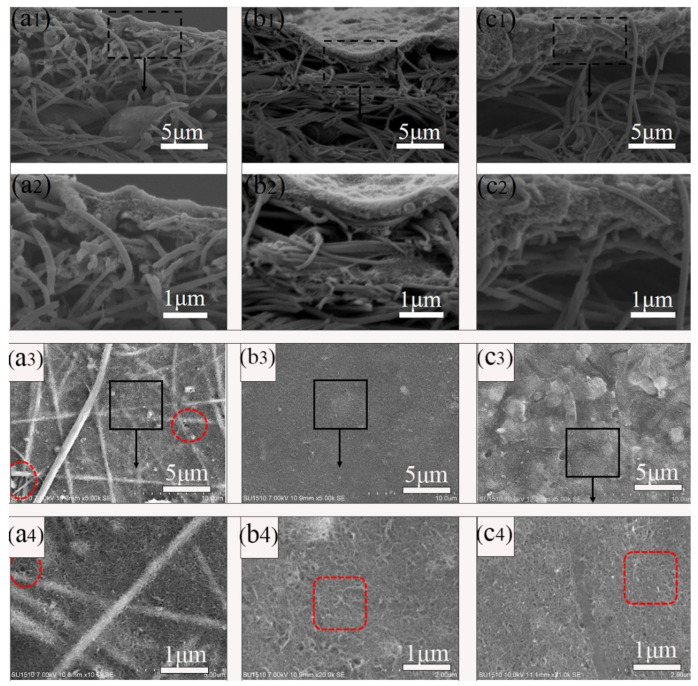
Cross-sectional and surface SEM images of PDA@MWCNTs/PVDF: (**a1**–**a4**) S-2, (**b1**–**b4**) S-3, (**c1**–**c4**) S-4.

The PDA@MWCNTs supernatant dispersion solution after centrifugal treatment was deposited on the nanofiber support layer. The PDA@MWCNTs thin layer obviously improved the pore structure and properties of the nanofiber membrane, such as the morphology, pore size, porosity, roughness, and hydrophilic ability. The surface and cross-section morphology of the PDA@MWCNTs/PVDF support layer are shown in Figure 2. The PDA@MWCNTs layer presents a continuous and porous network structure, and the surface pore size is relatively uniform. Compared to the tree-like nanofiber membrane (Supplementary Figure S1), the PDA@MWCNTs ultrathin film surface pore diameter from hundreds of nanometers decreases to tens of nanometers. The PDA@MWCNTs content significantly influences the surface morphology of the support layer. When the content is low, the PDA@MWCNTs does not effectively coat the nanofiber membrane surface. Some fiber structures can be observed, which might cause some defects. When the content is high, PDA@MWCNTs are excessively deposited on the surface, and surface pore size decreases.

The PDA@MWCNTs/PVDF composite support layer comprises the PVDF tree-like nanofiber membrane and PDA@MWCNTs thin film (Figure 2). The preparation process of the PVDF tree-like nanofiber membrane is detailed in our previous experimental work [29]. In this study, we fabricated three different composite support layers by changing the content of PDA@MWCNTs.

**Figure 3:**
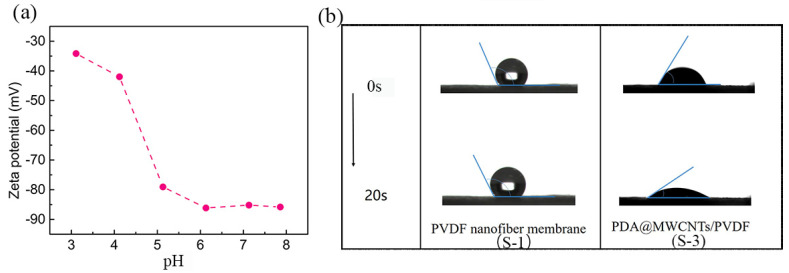
(**a**) Change in zeta potential at different pH values of S-3 membrane, (**b**) the surface dynamic contact angle change in PVDF nanofiber membrane and S-3 membrane.

The dynamic wettability of the optimal support layer and nanofiber membrane are shown in Figure 3b. The PVDF nanofiber has a hydrophobic nature which makes it difficult to wet. As is known to all, PDA is an excellent hydrophilic material. PDA attached to MWCNTs can effectively improve the surface hydrophilic of MWCNTs. The hydrophilic PDA@MWCNTs thin layer conduces to the formation of PA films via the interfacial polymerization reaction. All the support layer water contact angles are shown Supplementary Figure S2. Moreover, PDA possesses good adhesion results: the PDA@MWCNTs cannot fall off the nanofiber membrane. Furthermore, the resulting PDA@MWCNTs/PVDF membrane presented good thermal stability (Supplementary Figure S3); it can endure long-time operations at high water pressures.

The charge effect and PA film pore size are closely related to the salt ions’ rejection of the PA TFC membrane. The charge effect plays a leading role in screening ions. The zeta potential is used to characterize the charge of the support layer surface, and the change in the PDA@MWCNTs/PVDF membrane surface charge, as Figure 3a shows [30,31]. The PDA@MWCNTs film surface presents electronegativity at the range of 3–8 pH, which indicates that the support layer surface possesses a large amount of negative charge. The zeta potential value is 87 mV under neutral conditions. The PA film is negatively charged because of carboxyl groups arising from the acyl chloride hydrolysis. Therefore, the PA film formed on the PDA@MWCNTs/PVDF support layer possesses higher negatively potential. Under normal conditions, a film surface with higher zeta potential has a greater electrostatic repulsion effect on the same charged ion. Based on the above, we designed the PDA@MWCNTs/PVDF support layer to contribute to improving the separation properties of the PA TFC membrane.

### 2.3. The Morphology and Properties of PA Film

#### 2.3.1. Micromorphology

**Figure 4:**
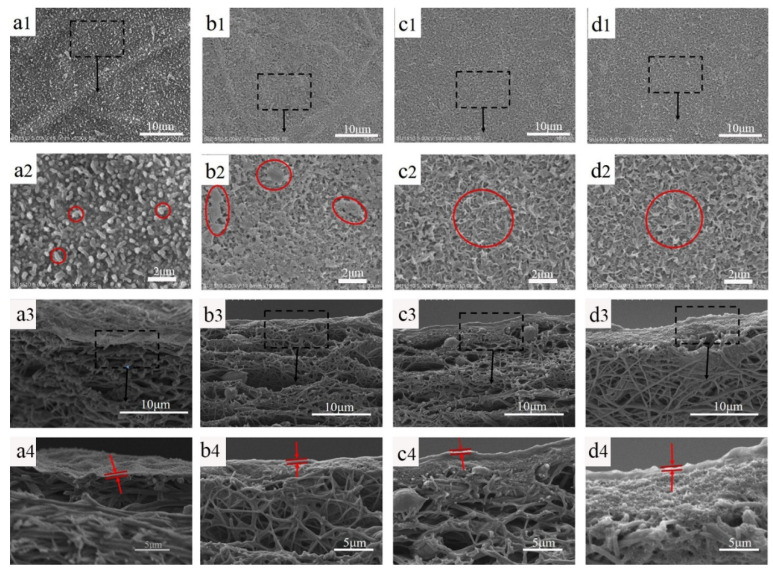
Surface and cross-sectional images of PA TFC membranes with different support layers, (**a1**–**a4**) TFC-1 (the black squares for local amplification), (**b1**–**b4**) TFC-2 (the red squares for PA surface), (**c1**–**c4**) TFC-3, (**d1**–**d4**) TFC-4 (the PA film between the red arrows).

PA TFC membranes with various support layers were prepared via the interfacial polymerization of MPD and TMC. Figure 4 shows the surface and cross-sectional morphology of the composite membranes. It is obvious that the PA film on the nanofiber membrane presents an obvious spherical protrusion structure, and the PA films with the PDA@MWCNTs support layer show a typical ridge-and-valley structure. This is because the nanofiber membrane with a large pore size and surface roughness slows the organic solution diffusion rate, making the formed PA film appear to have a nodular-based rough surface. PDA@MWCNTs support layers with a small and uniform pore size, as well as a smoother surface, quickens the reaction of MPD and TMC, and the nascent PA starts to appear turbulence as the reaction persists, forming the ridge-and-valley structure. However, the thickness of the PDA@MWCNTs layer significantly influences the complete morphology of the PA film. S-2 with the thinnest PDA@MWCNTs layer results in some defect sections of the PA film being formed. S-3 and S-4 with thicker PDA@MWCNTs layers contribute to a defect-free PA film being formed. A complete and defect-free PA film is one of the important guarantees of the TFC membrane with excellent separation properties.

#### 2.3.2. Separation Property

**Figure 5:**
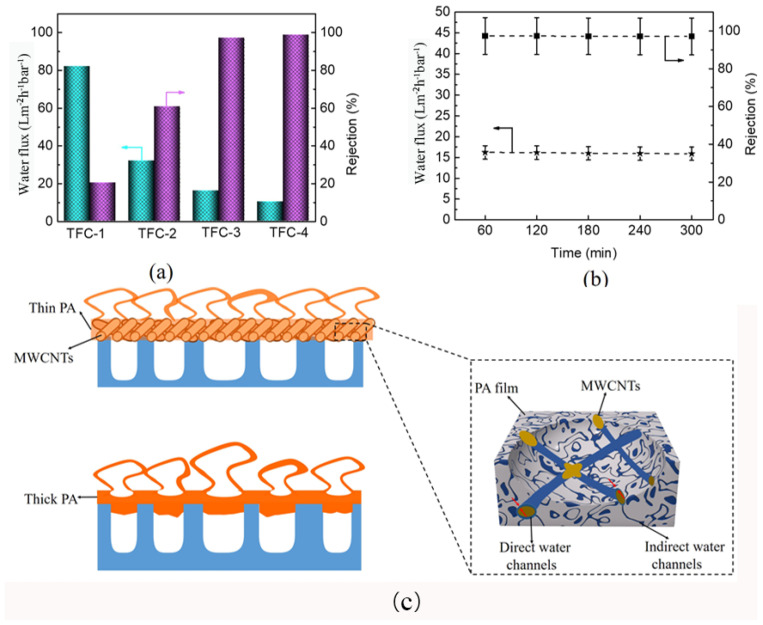
(**a**) Separation performance of various PA TFC membranes; (**b**) stability of PA TFC membrane with S-3 support layer for long-time operation; (**c**) forming mechanism of PA film with different support layers.

Water flux and salt ions rejection are the two key indicators to measure PA TFC membrane permselectivity. Figure 5a exhibits the fabricated PA TFC membrane’s water flux and rejection of the Na_2_SO_4_ solution. The TFC-1 possesses the highest water flux, but the salt rejection rate is the lowest. This is because the nanofiber membrane support layer with a large pore size easily causes PA film fractures at high pressures. Compared with TFC-1, TFC-2, TFC-3, and TFC-4 present higher salt ions rejection and lower water fluxes. However, the rejection of PA-2 is relatively low, which can be attributed to the defected PA film formed on S-2. Notably, TFC-3 and TFC-4 present similar rejections, but the water flux of TFC-4 is lower. This can be ascribed to the thicker PDA@MWCNTs layer storing more MPD aqueous solution, resulting in a thicker PA film being formed, which increased the water transmission distance and transmission resistance. The rejection behavior of TFC-3 to different salt solution is shown in Supplementary Figure S4. The composite membrane exhibited higher rejection to Na_2_SO_4_ and MgSO_4_. We have compared the separation performance of PA TFC membranes with other reports (Supplementary Table S1), we found that modifying the PA film can obtain higher water fluxes compared with optimizing the support layer. However, we obtained the highest water flux only by improving the support membrane property. To further determine the practice application performance, the optimal TFC-2 membrane was continuously operated for 300 min to analyze the stability. The result (Figure 4b) shows that the water flux and rejection of the PA TFC membrane almost remain unchanged.

#### 2.3.3. Mechanistic Insights

All the above analyses indicated that the PDA@MWCNTs layer significantly affects PA film performance. The detail mechanistic of action is depicted in Figure 5c. Tree-like nanofiber membranes with large pores can store more MPD aqueous solution, which is conductive to producing a thicker PA film and even a portion of PA film being inserted into the pores. Nonetheless, the large pore diameter of the support layer did not withstand high pressure at practical application and caused PA film damage. However, the PDA@MWCNTs layer not only could decrease the nanofiber membrane pore size but regulate the PA film thickness so that the resulting PA film shows excellent stability and separation [32,33]. Moreover, the new water channels between the PDA@MWCNTs and PA film accelerated the transport of water. Nonetheless, the PDA@MWCNTs content also played an important role. When the content was insufficient, the PDA@MWCNTs could not uniformly cover the top of the nanofiber membrane, causing a defect of the support layer surface. When the content was too much, a thicker PDA@MWCNTs layer caused a thicker PA film to be formed and resulted in a decrease in the flux.

## 3. Experimental Section

### 3.1. Materials

The polyvinylidene fluoride (PVDF) material was purchased from Solef, (Changzhou, China). N,N-dimethylformamide (DMF) and acetone were purchased from Sinopharm Chemical Reagent Co., Ltd. (Shanghai, China). Acid-treated MWCNTs powder (diameter: <15 nm, length: 0.5–2 μm; purity: >95%) was provided by XFNANO Technology Co., Ltd. (Nanjing, China). M-phenylenediamine (MPD) and trimethyl chloride (TMC) as monomers of the interfacial polymerization reaction were purchased from Damas-beta (Shanghai, China). Other chemicals used in our experiment were all purchased from Sigma-Aldrich (St. Louis, MO, USA).

### 3.2. Preparation of PDA@MWCNNTs

**Figure 6:**
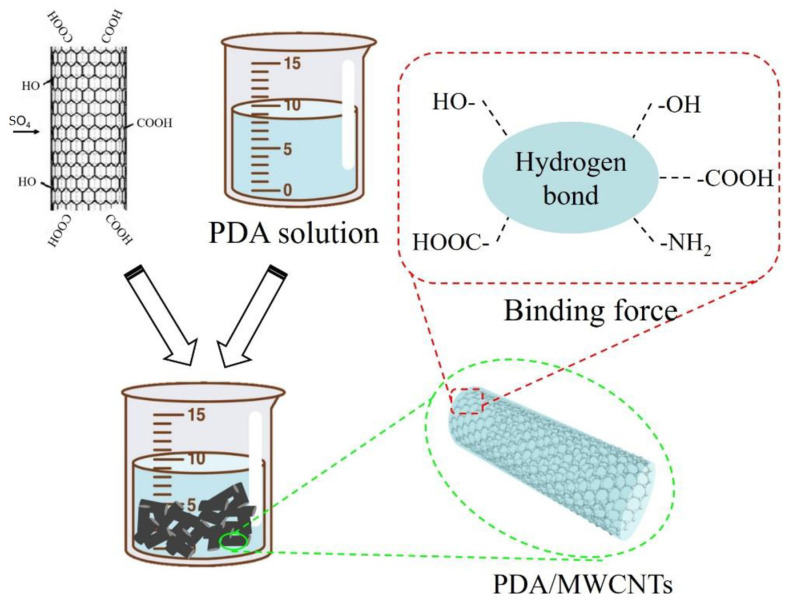
Mechanism diagram of PDA-modified acid-treated MWCNTs.

First, we prepared the Tris-HCl buffer solution with a concentration of 50 mM and a pH of 8.5. Next, we measured 100 mL buffer solution, 0.2 g polydopamine, 0.016 g CuSO_4_ solution, and 40 μL H_2_O_2_ solution which were added to the beaker, respectively. And, 1 g MWCNTs were added to the beaker for ultrasonic dispersion. Finally, the mixed solution was reacted for 1 h at 50 °C. The mechanism is shown in Figure 6.

### 3.3. Preparation of PDA@MWCNTs/PVDF Composite Support Membrane

**Figure 7:**
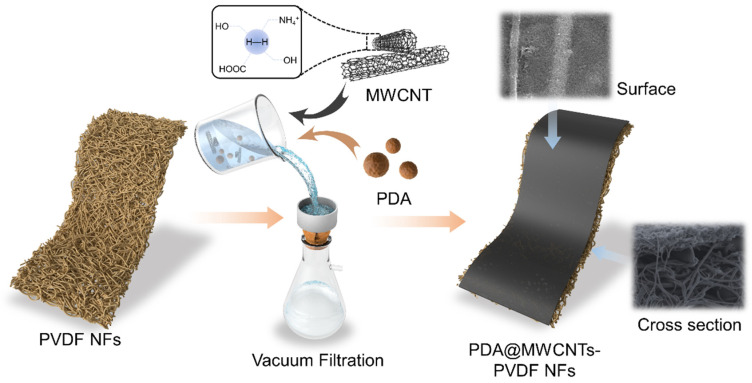
Forming mechanism of PDA@MWCNTs/PVDF support layer.

We measured a certain amount of the PDA@MWCNTs solution and sodium dodecylbenzenesulfonate (SDS) which were added to Milli-Q water and sonicated 10 h. Then, the dispersion was centrifuged at 10,000 rpm for 1 h to remove undispersed PDA@MWCNTs. The PDA@MWCNTs dispersion solution was further diluted and deposited on the nanofiber membrane surface via the vacuum filtration method. The detailed experimental process is shown in Figure 7. The resultant support layers were labeled S-2 to S-4, and S-1 was the control sample. The fabrication details of the nanofiber membrane was described in our previous work [26].

### 3.4. Preparation of PA TFC Membrane

The interfacial polymerization process is described in our previous work [34]. The PDA@MWCNTs/PVDF support layer was fixed on a glass plate. The MPD solution of 2 wt% was used to wet the PDA@MWCNTs thin film, and the residual solution was removed via air knife. Then, the TMC solution of 0.15 wt% was used to cover the surface to react for 2 min, and the excess solution was rinsed by acetone solution. The resultant composite membrane was treated in the oven at 70 °C for 10 min. Finally, the composite membranes were stored in DI water for further testing. The resultant PA TFC membranes were labeled as PA-1 to PA-4.

### 3.5. Characterization

#### 3.5.1. Microscopy Characterization via SEM and TEM Testing

The microscopy morphology of MWCNTs and the PA film was characterized via scan electron microscopy (SEM). The samples were dried in the oven at 60 °C before measuring. The morphology of the modified MWCNTs were analyzed via a transmission electron microscope (TEM).

#### 3.5.2. Membrane Separation Performance Evaluation

The salt ion rejection and water flux of the composite membranes were measured by a lab-scale device in a cross-flow system and an effective testing area of 3.14 cm^2^. The separation performance was tested at the operation pressure of 0.5 MPa. A Na_2_SO_4_ solution of 2000 ppm was the feed solution. The same film was measured at least 5 times to calculate the average. The water flux and rejection were calculated by the following equation [35,36].

(1) $$J=\frac{V}{A\times t}$$

where *J* is the water flux (L/(m^2^ h)), *t* is the test time (h), and *V* and *A* are the water volume (L) and the effective measure area (cm^2^), respectively.

(2) $$R=\left(1-\frac{Cp}{Cf}\right)\times 100\%$$

where *R* is the rejection ratio (%), and *Cp* and *Cf* are the conductivity of the permeate and feed (μs/cm), respectively.

#### 3.5.3. Zeta Potential Analysis

The surface zeta potential value of the membrane was tested by a zeta potential analyzer. The test pH range was 3–10, and the test solution was 1 mM potassium chloride solution. Before testing, the sample needed to be cut into 3 pieces × 3 cm size.

## 4. Conclusions

In this study, we designed and constructed a new support layer with a uniform surface pore structure and small pore size. The support layer was composed of PDA@MWCNTs and a PVDF tree-like nanofiber membrane. PDA is endowed with excellent dispersity and improves the hydrophilic capacity of MWCNTs. The uniformly dispersed PDA@MWCNTs were deposited on a tree-like nanofiber membrane surface via vacuum filtration. Regulating the PDA@MWCNTs film thickness not only optimized the surface structure of the nanofiber membrane but controlled the interfacial polymerization condition, obtaining a high-performance PA film. The resultant PA TFC membrane exhibited a water flux up to 15.8 L/(m^2^·h·bar) and a rejection rate of 97% to Na_2_SO_4_ solution at 0.5 MPa.

## Data Availability

Data are contained within the article and Supplementary Materials.

## Supplementary Materials

The following supporting information can be downloaded at: https://www.mdpi.com/article/10.3390/molecules29071460/s1, Figure S1: (a1,a2) Tree-like nanofiber membrane SEM images; Figure S2. The contact angle of various support layer; Figure S3. TG curves of PVDF nanofiber membrane and PDA@MWCNTs/PVDF membrane; Figure S4. The rejection of membrane TFC-3 to various salt solution; Table S1: Comparison of the result in this work with other results of report for Na_2_SO_4_ separation performance. References [6,31,37,38,39,40,41] are cited in the Supplementary Materials.
