# Supplementary material for: The Preparation and Performance Study of Polyamide Film Based on PDA@MWCNTs/PVDF Porous Support Layer
Source: Molecules. 2024 Mar 25;29(7):1460. doi: 10.3390/molecules29071460 (PMC11013461; doi:10.3390/molecules29071460)
Supplement: Supplementary file 1 [file molecules-29-01460-s001.zip › molecules-2834023-supplementary.pdf]

1. Supplementary figure

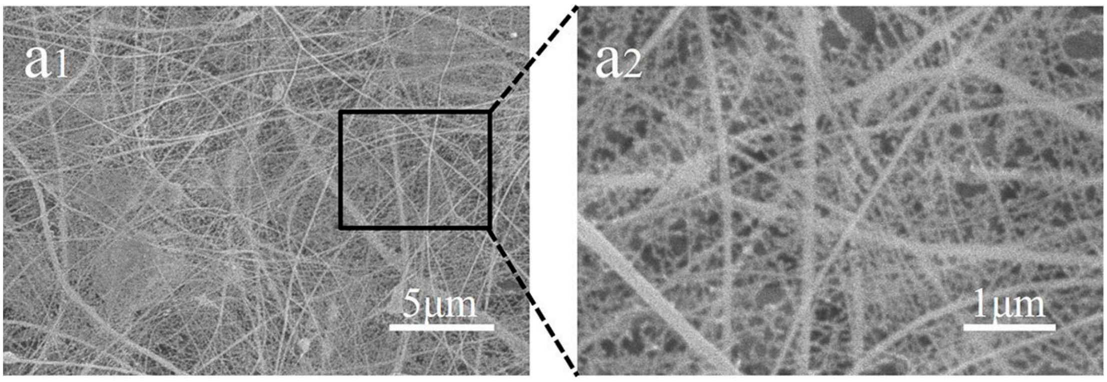

Supplementary **Figure S1.** (a1, a2) Tree-like nanofiber membrane SEM images.

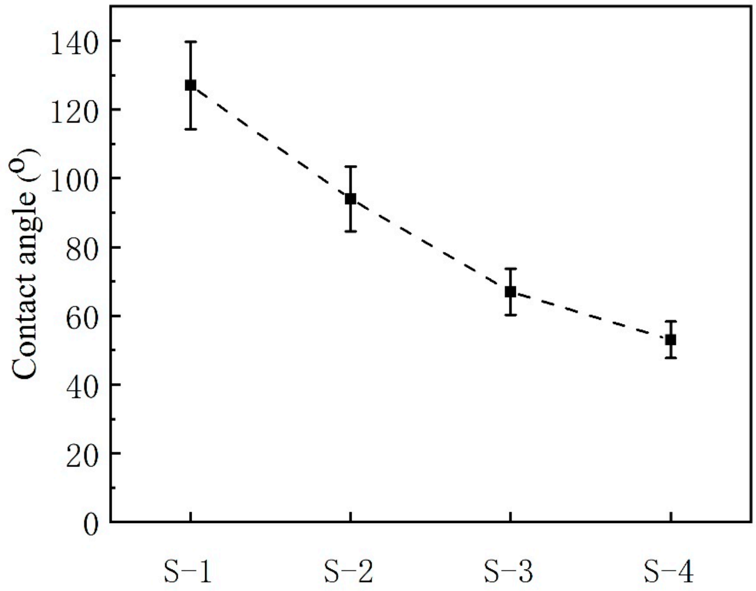

Supplementary **Figure S2**. The contact angle of various support layer.

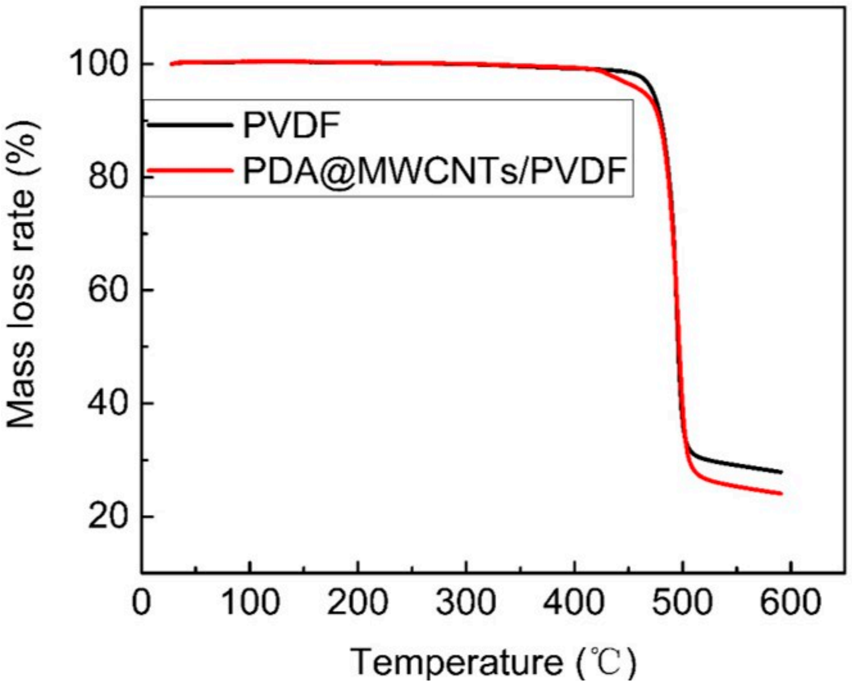

Supplementary **Figure S3.** TG curves of PVDF nanofiber membrane and PDA@MWCNTs/PVDF membrane.

The thermal stability of PA TFC membrane is a crucial indicator in practical application. The TG curves (Supplementary Figure S2) indicates the nanofiber membrane and PDA@MWCNTs membrane both keep excellent stability under 400 ^{o}C.

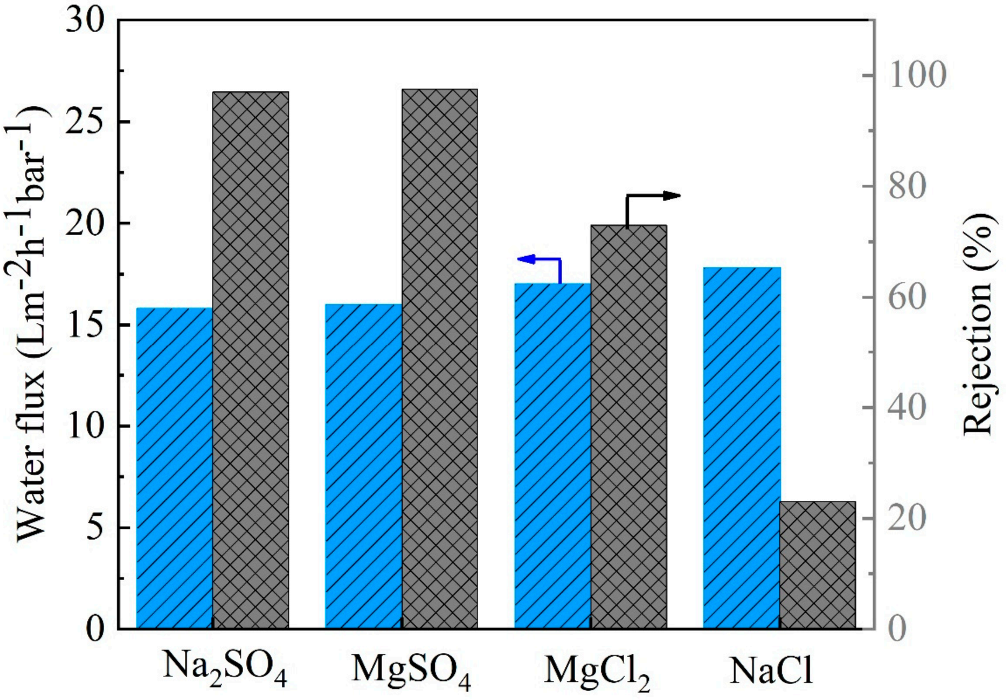

Supplementary **Figure S4**. The rejection of membrane TFC-3 to various salt solution.

Supplementary Table S1. Comparison of the result in this work with other results of report for Na_2SO_4 separation performance.

PA TFC membrane	Salt rejection (%)	Water flux (LMH bar^{-1})	Ref
MWCNTs/PSF support	75	2.33	[1]
CNTs/PA film	99.1	25.1	[2]
Modified-MWCNTs/PA film	99	6.98	[3]
Metal Organic Framework/PA film	65	39.5	[4]
Control PA film	97	17.17	[4]
Improve IP reaction	96	34.7	[5]
o-POPs/PA film	94.9	29.6	[6]
Reactable substrate	6.2	6.3	[7]
PDA@MWCNTs/PVDF Support	97	15.8	This work

References

[1] V. Vatanpour, S. S. Madaeni, R. Moradian, S. Zinadini, B. Astinchap, Fabrication and characterization of novel antifouling nanofiltration membrane prepared from oxidized multiwalled carbon nanotube/polyethersulfone nanocomposite, *J. Membr. Sci.* **2011**, 375, 284-294.

[2] J. Zhu, J. Hou, R. Zhang, S. Yuan, J. Li. M. Tian. P. Wang, Y. Zhang, A, Volodin and B. V. Bruggen. Rapid water transport through controllable,

ultrathin polyamide nanofilms for high performance nanofiltration, *J. Mater. Chem. A*. **2018**, 6, 15701-1509.

[3] J. Shen, C. Yu, H. Ruan, C. Gao, B. V. Bruggen. Preparation and characterization of thin-film nanocomposite membranes embedded with poly(methyl methacrylate) hydrophobic modified multiwalled carbon nanotubes by interfacial polymerization, *J. Membr. Sci*. **2013**, (442), 18-26.

[4] R. Dai, H, Guo, C. Tang, M. Chen, J. Li, Z. Wang. Hydrophilic Selective Nanochannels Created by Metal Organic Frameworks in Nanofiltration Membranes Enhance Rejection of Hydrophobic Endocrine-Disrupting Compounds, *Environ. Sci. Technol.* **2019**, 53, 13776–13783.

[5] Y. Liu, J. Zhu, J. Zheng, X. Gao, J. Wang, X. Wang, Y. F. Xie, X. Huang and B. V. Bruggen, A Facile and Scalable Fabrication Procedure for Thin-Film Composite Membranes: Integration of Phase Inversion and Interfacial Polymerization. *Environ. Sci. Technol.* **2020**, 54, 1946–1954.

[6] Ya. Liu, J. Zhu, J. Zheng, X. Gao, M. Tian, X. Wang, Y. F. Xie, Y. Zhang, A. Volodin and B. V. Bruggen, Porous organic polymer embedded thin-film nanocomposite membranes for enhanced nanofiltration performance, *J. Membr. Sci. 2020*, 602, 117982.

[7] Z. Yao, H. Guo, Z. Yang, C. Lin, B. Zhu, Y. Dong, C. Y. Tang, Reactable substrate participating interfacial polymerization for thin film composite membranes with enhanced salt rejection performance, *Desalination,* **2018**, 436, 1-7.
